# Short Latency Gray Matter Changes in Voxel-Based Morphometry following High Frequent Visual Stimulation

**DOI:** 10.1155/2017/1397801

**Published:** 2017-02-15

**Authors:** Steffen Naegel, Tim Hagenacker, Nina Theysohn, Hans-Christoph Diener, Zaza Katsarava, Mark Obermann, Dagny Holle

**Affiliations:** ^1^Department of Neurology, University Hospital Essen, University of Duisburg-Essen, Hufelandstr. 55, 45122 Essen, Germany; ^2^Department of Diagnostic and Interventional Radiology and Neuroradiology, University Hospital Essen, University of Duisburg-Essen, Hufelandstr. 55, 45122 Essen, Germany; ^3^Department of Neurology, EK Unna, Holbeinstr. 10, 59423 Unna, Germany; ^4^Center for Neurology, Asklepios Hospitals Schildautal, Karl-Herold-Straße 1, 38723 Seesen, Germany

## Abstract

Magnetic resonance imaging studies using voxel-based morphometry (VBM) detected structural changes in the human brain within periods of months or weeks. The underlying molecular mechanisms of VBM findings remain unresolved. We showed that simple visual stimulation by an alternating checkerboard leads to instant, short-lasting alterations of the primary and secondary visual cortex detected by VBM. The rapidness of occurrence (i.e., within 10 minutes) rather excludes most of the proposed physiological mechanism such as neural or glial cell genesis/degeneration or synapse turnover. We therefore favour cerebral fluid shifts to be the underlying correlate of the here observed VBM gray matter changes. Fast onset gray matter changes might be one important explanation for the inconsistency of VBM study results that often raise concern in regard to the validity of presented data. This study shows that changes detectable by VBM may occur within a few minutes after physiological stimulation and must be considered in future VBM experiments to avoid misinterpretation of results.

## 1. Introduction

Magnetic resonance imaging (MRI) voxel-based morphometry (VBM) studies of the human brain suggest detecting structural alterations of gray matter (GM) in response to external stimuli or states of disease. VBM is a whole brain technique capable of revealing subtle changes in GM by applying voxel wise statistics [1]. In the past years VBM has become a very popular method to describe GM structural changes in the human brain. Many different studies showed that diseases, training, or medication induces GM changes within weeks, months, or years. A few studies even detected earlier cerebral changes within 7 days in volunteers who learned to juggle [2] or within 5 days after repetitive transcranial magnetic stimulation of the superior temporal gyrus [3]. One study even demonstrated GM volume increase of the ventral putamen within a few hours after application of the D_2_ receptor blocker haloperidol [4].

Little is known about the genuine physiological changes that cause these alterations. Although VBM changes are always referred to as structural changes in contrast to functional changes reflected by functional MRI (fMRI) with blood oxygen level dependent (BOLD), the true nature of the visualized alterations remains enigmatic. Three possible mechanisms were proposed to explain VBM changes: (1) increase/decrease in cell size and neural or glial cell genesis/degeneration [6]; (2) variance in spine density [7, 8] and synapse turnover [8]; and (3) changes in blood flow or interstitial fluid [9].

The question of the underlying morphology and inconsistencies of some VBM studies from different working groups as well as partially insufficient data reproducibility raised concerns about the validity and correct interpretation of VBM studies in general [10]. According to current opinion these differences result predominantly from different MRI scanners and different sequence parameters used. This seems to be only half true as the full capacity of VBM analyses remains widely unexplored in spite of recent studies demonstrating GM changes within shorter and shorter time intervals. We aimed to stress this development to see whether we would be able to detect instant brain changes with the conventional VBM technique after visual stimulation with a flickering checkerboard for 10 minutes. Our objective was to determine whether short latency GM changes following simple visual stimulation would be detectable by VBM as potential new application for this technique and also whether this phenomenon could be a possible influence which must be controlled for in future studies with regard to result interpretation.

## 2. Material and Methods

### 2.1. Subjects and Stimulation Paradigm

All participants gave their written informed consent according to the Declaration of Helsinki prior to study inclusion. The local ethics committee of the University of Duisburg-Essen approved the study protocol. Twenty healthy subjects (11 women and 9 men; mean age: 27.8 years; range: 21–36 years) were investigated. The study was performed on a 1.5T scanner (Sonata, Siemens Healthcare, Germany) using a standard 8-channel birdcage head coil. The visual stimulus consisted of a 20 × 15 black and white checkerboard pattern with alternating squares flashing five times per second (5 Hz) for 10 minutes. The pattern was generated using Matlab® (The Mathworks, Inc., Natick, MA) and projected via beamer into the scanner room. Checkerboard stimulation (CS) was applied via mirror in supine position in the MRI scanner.

### 2.2. Voxel-Based Morphometry (VBM)

T1-weighted magnetization prepared rapid acquisition gradient echo (MPRAGE) sequence was acquired: TR/TE/TI = 2400 ms/4.38 ms/1200 ms, flip angle = 8°, field of view = 256 mm, 160 slices, and voxel size 1 × 1 × 1 mm^3^. Three MPRAGE sequences at three different time points (TP) were acquired of each subject: (1) before CS (TP 1); (2) right after CS (TP 2); (3) 60 minutes after CS (TP 3). Thirty minutes before the first scan and between the scans subjects waited in a quiet room without augmented external influence such as noise, smell, or excessive optical stimulation. Consumption of drinks and food was prohibited 3 hours before and during the entire experiment. Data processing and analysis were done with SPM8 (http://www.fil.ion.ucl.ac.uk/spm/) including New Segmentation and the “DARTEL” (Diffeomorphic Anatomical Registration Through Exponentiated Lie Algebra) algorithm using Matlab (Matlab 7, The MathWorks, Natick, MA, USA) [11]. Full factorial design (factor time, 3 levels: before CS, right after CS, and 60 minutes after CS) was used for statistical analysis. We entered all anatomical scans from all subjects into a single (fixed effects) general linear model. Note that each subject only brings two degrees of freedom to this analysis, rendering a mixed or random effects analysis unnecessary. Only results that were significant at *p* < 0.05 (FWE corrected) in the whole brain analysis are reported.

### 2.3. Functional MRI (fMRI)

Functional MRI (fMRI) was performed after completion of the VBM paradigm as control experiment to ensure proper activation of the visual cortex by the CS. A T_2_^*∗*^ weighted gradient echo planar imaging (EPI) sequence (TE = 55 ms, TR = 3200 ms, flip angle = 90°, acquisition time 3.95 minutes (74 scans), 34 transaxial slices (slice thickness = 3 mm), matrix 64 × 64, field of view 384 × 384 mm^2^, and voxel size 3.4 × 3.4 × 3.0 mm) was used in a block-design-sequence. The first four scans were discarded from fMRI-analysis to account for relaxation effects. The same checkerboard stimulation (CS) was applied in two “activation” epochs. In the “rest” epochs a black screen was presented. Each activation epoch lasted seven scans. Data processing and analysis were performed with SPM8 (http://www.fil.ion.ucl.ac.uk/spm/) using Matlab (Matlab 7, The MathWorks, Natick, MA, USA). Only results that were significant at *p* < 0.05 (FWE corrected) in the whole brain analysis are reported.

## 3. Results and Discussion

**Figure 1:**
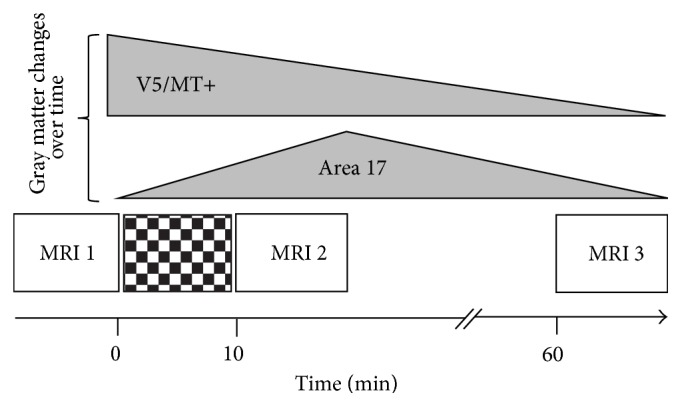
Study design and time course of gray matter changes. Schematic illustration of study design and time course of gray matter alterations. Looking 10 minutes at a flickering checkerboard leads to distinct changes of the cerebral gray matter detected by VBM. After 10 minutes an increase of the gray matter compartment can be observed in the primary visual cortex (BA 17), which diminishes again within 1 hour. A decrease of the gray matter is found in the secondary visual cortex (Area V5/MT) with a temporal shift after CS reflecting secondary processing mechanisms. VBM: voxel-based morphometry; BA: Brodmann area.

**Figure 2:**
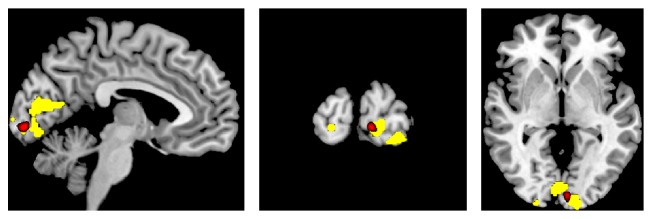
Structural alterations and functional activity following checkerboard stimulation. Functional MRI activation (yellow, MNI: *x* = −24, *y* = −94, *z* = −12; *p*_FWE_ < 0.001; *T* = 14.56) and VBM detected gray matter increase (red, MNI: *x* = −6, *y* = −93, *z* = −3; *p*_FWE_ = 0.043; *T* = 5.08) superimposed on skull striped anatomical (T1) image. GM increase and maximum BOLD signal are closely located in the primary visual cortex.

Study design and schematized results are shown in Figure 1. Directly after CS (TP2) a GM increase in the left primary visual cortex could be detected (BA17; MNI: *x* = −6, *y* = −93, *z* = −3; *p*_FWE_ = 0.043; *T* = 5.08). These alterations were reversed completely within 60 minutes (TP3). The GM increase was located close to the maximum BOLD signal chance in the primary visual cortex during the functional session (BA17; MNI: *x* = −24, *y* = −94, *z* = −12; *p*_FWE_ < 0.001; *T* = 14.56) but not identical (Figure 2). After 60 minutes (TP3) a decrease of GM in the right secondary visual cortex was detected (Area V5/MT; MNI: *x* = 59, *y* = −63, *z* = 7; *p*_FWE_ = 0.035; *T* = 5.19). Functional activity (yellow) and VBM detected GM changes (red) are both located in the same anatomic area, but the exact localization is slightly different between functional and structural imaging.

As expected, CS leads to a strong functional activity of the visual cortex, which was demonstrated in many previous studies (e.g., [12]). We were able to show that very similar changes within BA17 are also detectable using VBM after only 10 minutes of visual stimulation and diminish within the following hour. Further VBM changes of V5/MT can be shown one hour after stimulation.

The visual cortex is distinguishable into a primary visual cortex (striate cortex or V1; BA 17) and a secondary visual cortex (extrastriate cortical areas including V2, V3, V4, and V5; BA 18 and BA 19). V1 plays a crucial role in visual information processing and acts as a kind of “gatekeeper” for arriving visual stimuli. V5/MT is mainly involved in the perception of motion and is connected to a wide array of cortical and subcortical brain areas [13]. It is proposed that V1 contributes the most important input to V5/MT [13]. This strong connection, especially the distinct connection in series, might explain our observation of early VBM changes in V1 followed by changes in V5/MT.

The early VBM changes especially of the primary visual cortex develop within 10 minutes and completely resolve within 60 minutes suggesting the presence of fast-adapting cellular mechanisms in response to our stimulus. Whether these are early stages of cortical plasticity and whether the current understanding of structural brain changes as correlate of VBM analyses can be maintained remains unclear. Our results once again raise the question of the underlying molecular mechanisms of VBM and offer a new perspective on this problem. The rapid time course and reversibility of the observed changes excludes long lasting mechanisms such as cell or synapse genesis and favours the hypothesis that fluid shifts within the brain parenchyma might be the physiological correlate of short-term cortical plasticity, which allows to accomplish this fast adjustment of the brain or to be a consequence thereof.

Astroglial syncytium would be the most likely tissue responsible for accomplishing these fluid shifts as astrocytes have the capacity to preserve ion composition and volume of the peri- and intraneuronal space [14]. They are known to be involved in neurotransmitter modulation [15], homeostatic maintenance [16], neuronal energy supply [17], and neuronal development and regeneration [18]. A crucial role of astrocytes for brain function was reported in several diseases such as neuropathic pain [19], epilepsy [20], Alzheimer's disease [21], schizophrenia [22], and depression [23]. It was shown that substantial local shrinkage of the extracellular space (ECS) up to 30% occurs 10–20 seconds after neuronal activation under experimental conditions [24–30]. It was proposed that this ECS shrinkage phenomenon is based on combined potassium and water transport mechanisms. Exited neurons leak potassium ions to the ECS [31]; these are incorporated by the surrounding astrocytes if the neuronal firing frequency is increased [26, 31]. In turn of the potassium influx an osmolarity gradient is established that leads to water shift from the ECS into the astrocyte and leads to an increase or decrease of neuronal excitability to preserve homeostatic equilibrium and prevent cellular damage [27, 32]. In line with this hypothesis microscopic studies detected an increase of tissue due to water-induced swelling [33, 34]. Direct changes in tissue microstructure caused by pathophysiological neuronal activation during status epilepticus were visualized by diffusion MRI [35]. A transient decrease in the apparent diffusion coefficient (ADC) of water in the healthy human brain visual cortex was observed during stimulation with a black and white flickering checkerboard [36]. Although the decrease was only small (<1%), the change was observed in several voxels located within the BOLD activation area used as control condition and followed the time course of fMRI activation. The authors concluded that the ADC changes were ascribed to transient swelling of cortical cells [37]. This is very similar to our VBM data. Different authors discussed whether these diffusion-based changes of the working brain rather reflect residual long-latency vascular effects [38–40] or a combination of cell swelling related neural activation and vascular effects. Flint et al. [41] tried to further illuminate this questions by investigating changes in diffusion weighted MR-signals of a live hippocampus brain slice model after exposure to several substances (potassium, kainite, 6-cyano-7-nitroquinoxaline-2,3-dione, and MK-801). This experimental setup allowed recording of neuronal swelling without any interference from vascular effects. These observations are therefore more directly linked to neuronal events, in contrast to BOLD effects, which are considered to mainly reflect neurovascular changes. This might be an explanation why our V1 functional MRI activation was localized in slightly different anatomic area than our V1 VBM changes, but these differences may also be due to the different spatial resolution of both techniques as mentioned above. Further studies using SEEP (signal enhanced by extravascular protons) fMRI supported the observation that water shift induced shrinking or swelling of cells is accompanied by decrease or increase of MRI signal intensity mainly based on proton-density changes and do not solely result from the BOLD effect [42, 43].

VBM is an automated technique intended to assess structural changes in the brain. T1-weighted volumetric MRI scans are used for a statistical test across all voxels in the image in order to identify volume differences between several subject groups [1]. “Unified Segmentation” combines spatial normalisation and tissue classification into gray matter (GM), white matter (WM), cerebrospinal fluid (CSF), and three other background classes (“New Segmentation”). Classification is based on a mixture of Gaussians model (MOG) which represents the intensity probability density of MRI depicted tissue by a number of Gaussian distribution. It is still unsolved how these tissue classifications are related to the real cytoarchitecture of the brain, although almost all authors refer to observed GM changes as true morphological alterations. Our results show GM changes, meaning that more or less voxels are classified as this tissue class after looking at a flickering checkerboard. We assume that water shifts within the brain lead to voxel transition from one tissue class to another or alternatively lead to an increase/decrease in density of the predefined voxel due these water shifts. The rapidness of the occurrence of GM changes excludes genuine morphological changes in terms of a cytoarchitectonic increase of GM in our view.

**Figure 3:**
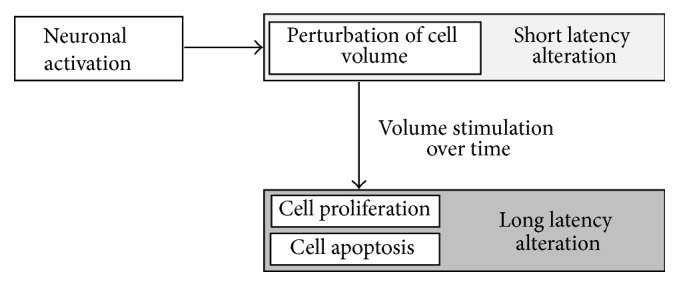
Possible physiological connection between short latency and long-latency voxel-based morphometry (VBM) alterations based on water shifts. Neuronal activation following flickering checkerboard stimulation goes along with immediate water depended changes of cell volume that could be detected as short latency gray matter changes in VBM paradigms. Persistent water alterations induce long-term downstream effects leading to cell proliferation and apoptosis that might reflect the long-latency gray matter changes in VBM paradigms (for review [5]).

Although the observed early GM changes might not explain the molecular mechanisms underlying VBM in all its complexity, they seem to represent an early phase of cortical changes that might provide one plausible explanation for the occasionally criticized inconsistency of VBM results. The question at which time point these obviously reversible, most likely fluid shift related changes convert into permanent localized structural changes that were reported in neurodegenerative disease and confirmed by neuropathology remains unresolved [44]. Whether these early changes are based on the same or at least similar mechanisms as the late onset changes also remains unclear. It could be hypothesized that different mechanisms (probably even determined by the underlying disease) account for early and late GM changes in VBM. However, it is well known that volume perturbations can induce long-term downstream effects such as proliferation, migration, and apoptosis (for review [5]), which might be the physiological link between short latency and long-latency VBM alterations (Figure 3).

It has to be noted that our experimental design confounded visual stimulation with the order of scan acquisition. In other words, the scans after photic stimulation were always acquired within a few minutes after the scans without photic stimulation. Strictly speaking, if we want to attribute the changes in apparent gray matter density to neuronally mediated visual stimulation, as opposed to a subsequent scanner effect, one would need a balanced longitudinal study that included conditions with and without visual stimulation. But, as the observed alterations are restricted to the visual system, we think it is highly unlikely that our results can be explained by nonspecific subsequent scanning effects.

In terms of the implications for standard VBM studies, our results suggest that there is a potential source of variability in gray matter density estimates used in VBM. In other words, the experience of subjects immediately prior to scanning may contribute a source of variability in longitudinal and group studies that could reduce the sensitivity (or reproducibility) of standard VBM studies. As the observed effect is small in amplitude and it is unlikely that there were systematic differences in intense prescanning experiences between groups or over time in previously reported cross-sectional and longitudinal studies, we are not generally questioning the validity of these but propose the inclusion of a resting period prior scanning in future VBM paradigms scanning to suppress short-lace latency effects of the sort we have reported.

## 4. Conclusion

The present study shows that stimuli-induced morphological changes occurring at the cellular level can be detected using VBM and that these changes are most likely related to extra- and intracellular fluid shifts. This might offer new scientific opportunities to apply the VBM technique in different experimental conditions that are not suitable for fMRI designs but require longer or psychopharmacological studies that have protracted effects on cerebral activity (or perfusion). Additionally, acute VBM might allow investigating secondary cerebral changes that occur within periods of minutes or hours after stimulation and which are not detectable for fMRI, which only shows immediate BOLD signal changes. Important consequence for future VBM studies is to control for these early changes, since experiment independent activity, for example, watching TV or reading a book in the waiting room, might introduce (short latency) variability into subsequent gray matter estimates and thereby reduce the efficiency of VBM paradigms and even may lead to misinterpretation.
